# The dinoflagellates *Durinskia baltica *and *Kryptoperidinium foliaceum *retain functionally overlapping mitochondria from two evolutionarily distinct lineages

**DOI:** 10.1186/1471-2148-7-172

**Published:** 2007-09-24

**Authors:** Behzad Imanian, Patrick J Keeling

**Affiliations:** 1 Canadian Institute for Advanced Research, Department of Botany, University of British Columbia, 3529-6270 University Boulevard, Vancouver, British Columbia V6T 1Z4, Canada

## Abstract

**Background:**

The dinoflagellates *Durinskia baltica *and *Kryptoperidinium foliaceum *are distinguished by the presence of a tertiary plastid derived from a diatom endosymbiont. The diatom is fully integrated with the host cell cycle and is so altered in structure as to be difficult to recognize it as a diatom, and yet it retains a number of features normally lost in tertiary and secondary endosymbionts, most notably mitochondria. The dinoflagellate host is also reported to retain mitochondrion-like structures, making these cells unique in retaining two evolutionarily distinct mitochondria. This redundancy raises the question of whether the organelles share any functions in common or have distributed functions between them.

**Results:**

We show that both host and endosymbiont mitochondrial genomes encode genes for electron transport proteins. We have characterized cytochrome c oxidase 1 (*cox1*), cytochrome oxidase 2 (*cox2*), cytochrome oxidase 3 (*cox3*), cytochrome b (*cob*), and large subunit of ribosomal RNA (*LSUrRNA*) of endosymbiont mitochondrial ancestry, and *cox1 *and *cob *of host mitochondrial ancestry. We show that all genes are transcribed and that those ascribed to the host mitochondrial genome are extensively edited at the RNA level, as expected for a dinoflagellate mitochondrion-encoded gene. We also found evidence for extensive recombination in the host mitochondrial genes and that recombination products are also transcribed, as expected for a dinoflagellate.

**Conclusion:**

*Durinskia baltica *and *K. foliaceum *retain two mitochondria from evolutionarily distinct lineages, and the functions of these organelles are at least partially overlapping, since both express genes for proteins in electron transport.

## Background

The endosymbiotic origins of plastids and mitochondria share a number of characteristics in common, [1,2], but differ in the complexity of their evolutionary history following their origin and initial integration. Whereas mitochondria originated once and have apparently never been lost [3-5], plastids have spread between eukaryotic lineages several times in events referred to as secondary and tertiary endosymbioses. Generally these secondary and tertiary endosymbionts have degenerated so far that all that remains is a plastid with extra membranes [6], but in a few exceptional cases intermediate stages of reduction are known, and these may provide interesting glimpses into how complexity is lost.

One of the characters that is absent from nearly all known examples of secondary and tertiary endosymbionts is the mitochondrion. This contrasts with the fact that mitochondria have never been lost in any other eukaryotic lineage. Even in the most severely reduced, anaerobic parasites which lack oxidative phosphorylation, highly reduced organelles called mitosomes and hydrogenosomes are found [3-5]. Some of these have no direct role in energy metabolism, but iron-sulfur cluster biosynthesis is a common function [5,7,8]. These relict organelles suggest mitochondria are resistant to outright loss, raising questions about why mitochondria appear to be one of the more dispensable features of algae taken up during secondary and tertiary endosymbiosis events.

The single clear exception to this is found in a group of related dinoflagellates that harbour a diatom tertiary endosymbiont. This group contains several species (see [9] for a recent summary), and here we have examined two: *Durinskia baltica *[10] and *Kryptoperidinium foliaceum *[11,12]. Several of these genera (including *Durinskia *and *Kryptoperidinium*) have been shown to share a common pennate diatom endosymbiont, arguing that the endosymbiosis is stable through evolutionary time [13,14]. Interestingly, this may not hold for the whole group, since the endosymbiont of *Peridinium quinquecorne *is a centric diatom [15], suggesting that the integration may have spanned a long period of time and different transient endosymbionts were ultimately fixed in these two subgroups. Nevertheless, the endosymbionts of *D. baltica *and *K. foliaceum *are no longer transient in the short term, they have lost motility and cell wall and, although some chromatin condensation occurs during sexual reproduction in *D. baltica*, typical chromosomes are not found within the endosymbiont nucleus at any stage of its life cycle [16,17]. During the endosymbiotic nuclear division, neither a spindle apparatus nor any microtubules have been observed [18], and the amitotic division of this nucleus results in unequal daughter nuclei and significantly larger amount of DNA in the nucleus than that reported in other diatoms [19].

The endosymbiont has clearly been reduced in many ways, but some of its most interesting characteristics are what it has retained. This includes plastids surrounded by endoplasmic reticulum (ER) that is continuous with the outer membrane of the nucleus, a plasma membrane that separates it from the host cytoplasm, a multi-lobed, prominent nucleus with a genome, ribosomes, dictyosomes, and mitochondria [16,20].

It is the retention of mitochondria that makes this endosymbiont stand out, in particular since *D. baltica *and *K. foliaceum *host cells have also been reported to retain mitochondria. The loss of endosymbiont mitochondria in virtually all known examples of secondary and tertiary endosymbiotic events suggests retaining two mitochondria is either unnecessary or even deleterious. The loss of one of these organelles may be ongoing, but it is also possible that both compartments require mitochondrial function or that they have distributed essential functions between them. We have previously shown [9] that the *K. foliaceum *endosymbiont mitochondrion contains a genome and expresses genes for cytochrome c oxidase subunit 1 (*cox1*), cytochrome c oxidase subunit 3 (*cox3*), and cytochrome b (*cob*). However, no data are available from the host mitochondrion, and with the function of this organelle completely unknown we cannot address the possibility that the two organelles have overlapping or differentiated function.

In order to determine whether this unique mitochondrial redundancy extends to the functional level, we characterized seven mitochondrial genes of *D. baltica*: five from the endosymbiont (*cox1*, *cox2*, *cox3*, *cob*, and *LSUrRNA*) and two from the host (*cox1 *and *cob*), and confirmed that *cox2 *and *LSUrRNA *from the endosymbiont and *cox1 *from the host are also present in *K. foliaceum*. Most significantly, in *D. baltica*, *cox1 *and *cob *are present and expressed in both mitochondria, and those in the host are heavily edited, as expected for a functional dinoflagellate mitochondrial gene [21-23]. All available data therefore suggest that both the host and endosymbiont mitochondria are actively expressing genes functional in oxidative phosphorylation and energy production.

## Results and discussion

### Characterization of endosymbiont mitochondrial genes and transcripts

PCR amplification using diatom-specific primers and total *D. baltica *DNA (or RNA, see below), resulted in fragments of the expected sizes for five genes: *cox1*, *cox3*, *cob*, *cox2*, and *LSUrRNA*. Sequencing multiple clones yielded a single copy of each gene. Only a short fragment of *cox1 *could be amplified from DNA, probably due to the presence of long type II introns such as those found in diatoms *Phaeodactylum tricornutum *and *Thalassiosira pseudonana *and the endosymbiont of *K. foliaceum *[9], so the remainder was recovered from RNA by RT-PCR. Diatom-derived genes for *cox1*, *cox3 *and *cob *are already known from *K. foliaceum *[9], so to complement the *D. baltica *data we also amplified the *K. foliaceum cox2 *and *LSUrRNA*.

**Figure 1:**
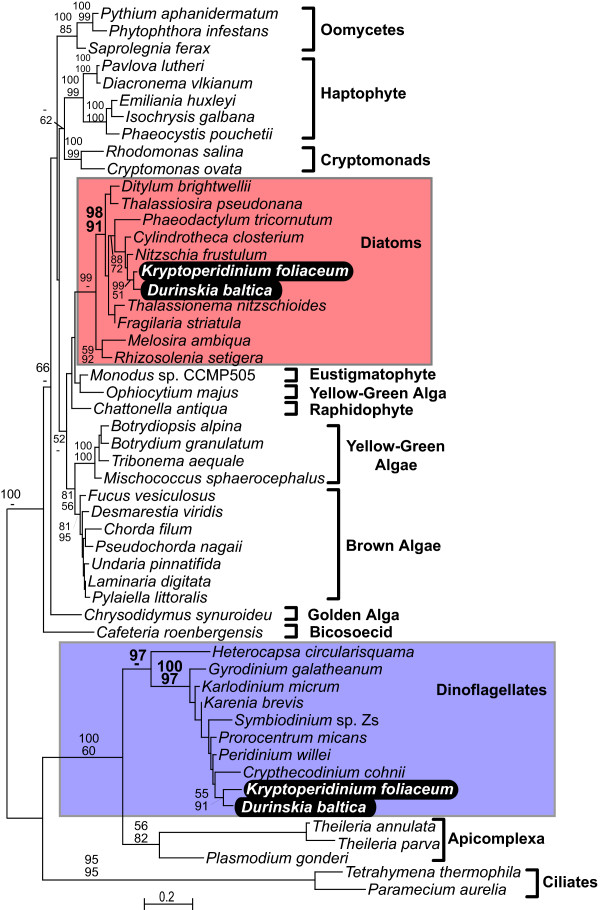
**Protein maximum likelihood phylogeny of cytochrome c oxidase 1 (*cox1*)**. Numbers at nodes indicate bootstrap support for major nodes over 50% from ML (top) and distance (bottom). A dash (-) indicates support less than 50%. Major groups are labeled to the right, with diatoms (red) and dinoflagellates (purple) indicated by a box and *D. baltica *and *K. foliaceum *genes in black.

**Figure 2:**
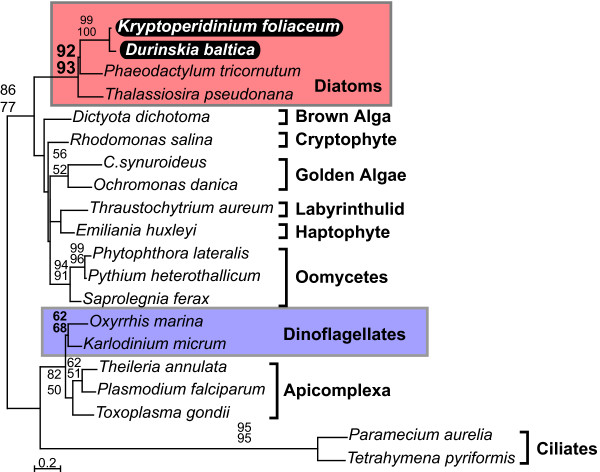
**Protein maximum likelihood phylogeny of cytochrome oxidase 2 (*cox2*)**. Numbers at nodes indicate bootstrap support for major nodes over 50% from ML (top) and distance (bottom). A dash (-) indicates support less than 50%. Major groups are labeled to the right, with diatoms (red) and dinoflagellates (purple) indicated by a box and *D. baltica *and *K. foliaceum *genes in black.

**Figure 3:**
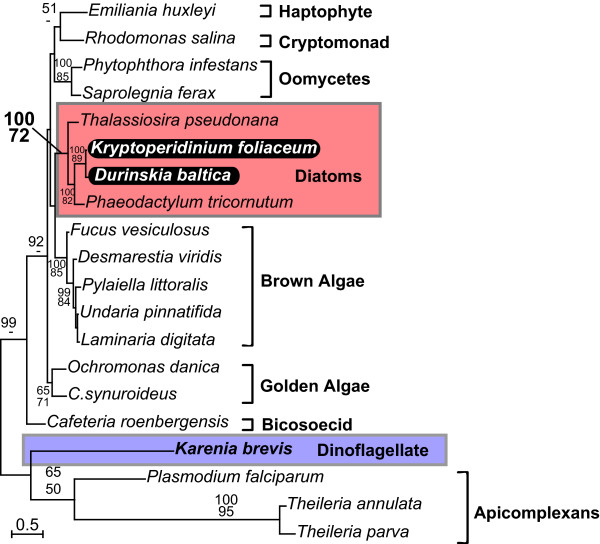
**Protein maximum likelihood phylogeny of cytochrome oxidase 3 (*cox3*)**. Numbers at nodes indicate bootstrap support for major nodes over 50% from ML (top) and distance (bottom). A dash (-) indicates support less than 50%. Major groups are labeled to the right, with diatoms (red) and dinoflagellates (purple) indicated by a box and *D. baltica *and *K. foliaceum *genes in black.

**Figure 4:**
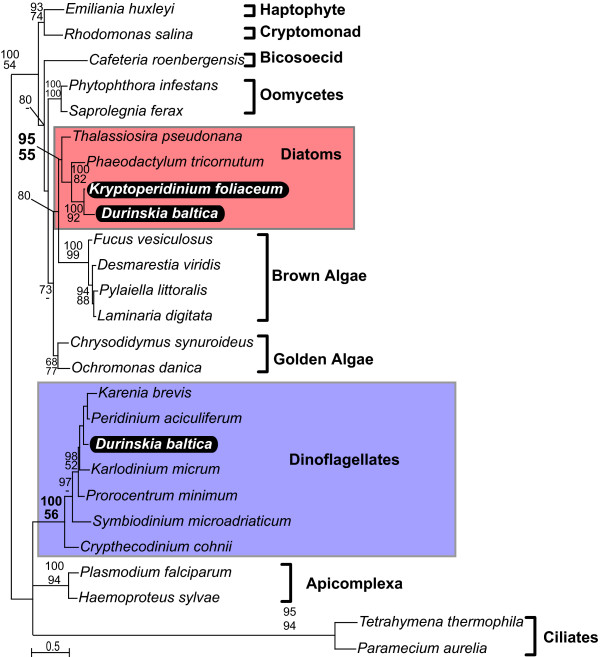
**Protein maximum likelihood phylogeny of cytochrome b (*cob*)**. Numbers at nodes indicate bootstrap support for major nodes over 50% from ML (top) and distance (bottom). A dash (-) indicates support less than 50%. Major groups are labeled to the right, with diatoms (red) and dinoflagellates (purple) indicated by a box and *D. baltica *and *K. foliaceum *genes in black.

**Figure 5:**
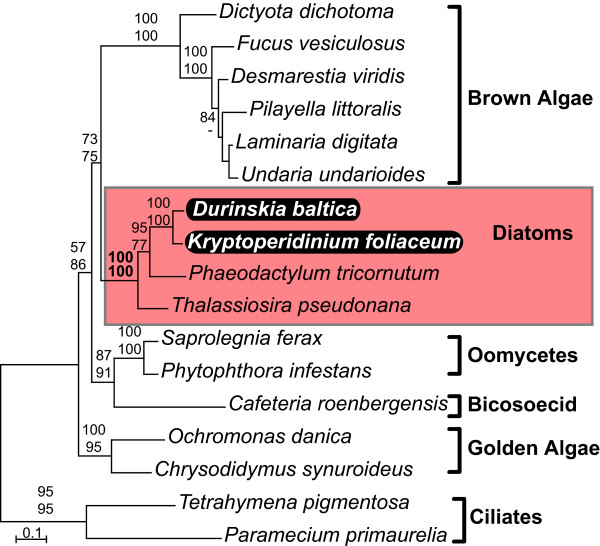
**Maximum likelihood phylogeny of large subunit ribosomal RNA (*LSUrRNA*) under General Time-Reversible (GTR) model of substitution**. Numbers at nodes indicate bootstrap support for major nodes over 50% from ML (top) and distance (bottom). A dash (-) indicates support less than 50%. Major groups are labeled to the right, with diatoms (red) indicated by a box and *D. baltica *and *K. foliaceum *genes in black.

The phylogenies of all five genes (Fig. 1, 2, 3, 4, 5) generally resembled trees based on nuclear genes, with relatively strong support for the monophyly of alveolates and sister relationship between dinoflagellates and apicomplexans [24,25]. We also noted that haptophytes and cryptophytes are sister groups with strong support in *cob *trees (Fig. 4) and weakly so in *cox1 *and *cox3 *trees (Fig. 1 and 3), as has been suggested in other analyses [26-29]. Most importantly, however, in all five phylogenies the distinction between the expected positions of host and endosymbiont-derived genes was unambiguous, and in all five trees the *D. baltica *gene amplified with diatom-specific primers branched within the diatom clade with strong support (Fig. 1, 2, 3, 4, 5). Moreover, *D. baltica *consistently grouped with *K. foliaceum *with strong support (with the exception of distance analysis of *cox1*, where the overlap between the two sequences is relatively short). This is in disagreement with the proposal that these dinoflagellates are products of separate endosymbiotic events [30], but consistent with the analyses of nuclear small-subunit rRNA genes from the hosts in these two dinoflagellates [14] and the hypothesis that these two species, and most likely their other close relatives, resulted from a single endosymbiotic event [13,15,31,32]. Together, *D. baltica *and *K. foliaceum *branched specifically with pennate diatoms (i.e. *Nitzschia *or *Cylindrotheca*, *Phaodactylum*), which is also consistent with evidence that the endosymbiont is a descendent of a pennate diatom [9,14,33,34]. Overall, these trees strongly support the conclusion that *cox1*, *cox3*, *cob*, *cox2*, and *LSUrRNA *are all present in the mitochondrial genome of the endosymbiont.

To confirm that all five genes are actively expressed, each was also amplified from RNA using RT-PCR. All diatom-derived cDNA sequences were identical to their corresponding genes, providing further evidence for these genes being from the endosymbiont mitochondrion, because the mitochondrial transcripts in dinoflagellates are extensively edited [22,35,36].

### Characterization of host mitochondrial genes and transcripts

**Figure 6:**
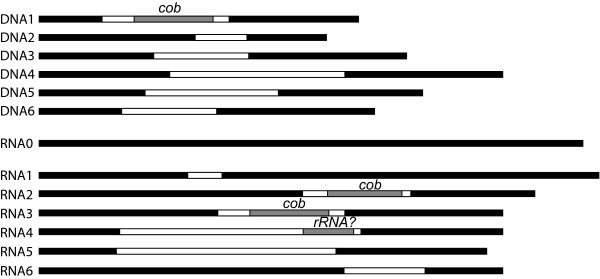
**Schematic representation of *cox1 *gene and cDNA fragments characterised from *Durinskia baltica***. The black rectangles represent coding regions of the gene or the transcript. The white rectangles represent the inserts. The inserts that contain a fragment of another gene have been represented by gray rectangles. The scale is proportional to the number of nucleotides.

The presence and expression of *cox *and *cob *genes in the endosymbiont mitochondria suggests this organelle is engaged in electron transport. The pressing question is therefore the nature of the host organelle, but no data have been gathered from it for comparison. Dinoflagellate mitochondrial genomes only encode three protein-coding genes: *cox1*, *cox3*, and *cob *[23]. The LSU rRNA has been extensively fragmented and rearranged [37], and cox2 has been split and moved to the nucleus [38], so only *cox1*, *cox3*, and *cob *were sought in the mitochondrial genome. We used dinoflagellate-specific primers for all three genes in RT-PCR with total *D. baltica *RNA, and identified fragments of *cox1 *and *cob*. Using 3' RACE, we also recovered the 3' end of the *D. baltica cob *gene. We also recovered 6 additional copies of *cox1 *transcripts (Fig. 6), each of which contained inserts that differed in position, size, and sequences, and disrupted the reading frame. Inserts ranged from 81 to 453 bp. Two inserts at slightly different positions of RNA2 and RNA3 were similar in size and sequences, and both of these contained a 151 bp portion of the *cob *gene flanked by two small (about 20 bp) non-coding fragments (Fig. 6). Another insert contained a 75 bp fragment with over 90% identity to non-coding fragments from the dinoflagellates *Alexandrium catenella *(Genbank accession: AB265207) *Gonyaulax polyedra *(Genbank accession: AF142472). We did not sequence a DNA clone lacking an insert, but the mitochondrial genome of other dinoflagellates is known to contain many copies of each gene and many rearrangements [21,23], so the intact copy of the gene was most likely simply not sampled. Given the highly fragmented and divergent nature of dinoflagellate mitochondrial rRNAs [37], it is possible this 75 bp represents an as yet unidentified fragment of either the LSU or SSU.

PCR using genomic DNA from *D. baltica *resulted in a single *cob *gene fragment (12 identical clones were sequenced), and six different *cox1 *fragments (seven different clones were sequenced). As with cDNAs, each *cox1 *gene contained a unique insert (Fig. 6). Most inserts were unique, but many contained small imperfect repeats in common, and the positions of the inserts within *cox1 *were variable and inevitably a portion of *cox1 *was missing at the point of insertion. One pair of DNA and cDNA clones (Fig. 6, DNA1 and RNA3) were found to be identical, with the exception of edited sites (see below).

A single transcript of the *K. foliaceum cox1 *was also recovered from sequencing 15 identical clones. This transcript also contained an insert, but without sequence similarity to anything known and not at a position in common with any *D. baltica *clone. We failed to identify *cob*, but considering its presence in *D. baltica *we feel it is unlikely to be absent in *K. foliaceum*.

The many variants of the *D. baltica *host *cox1 *gene and the insert in the *K. foliaceum cox1 *gene are both consistent with the nature of mitochondrial genomes in other dinoflagellates. This has been best described in *Crypthecodinium cohni *and *Amphidinium carterae*, where protein-coding genes are flanked by non-coding, repeat-rich sequences and that the context of a gene can vary in different copies due to homologous recombination [21,39], and in *Oxyrrhis marina *where protein coding genes are found in different genomic contexts, and are often fragmented [23].

### Host mitochondrial transcripts are extensively edited

Dinoflagellate mitochondria possess a distinctive form of RNA editing. Editing sites typically involve A to G, T to C, and C to U changes at first and second positions, affecting about 2% of positions in *cox1 *and *cob *genes [22,35,40]. The presence of such editing would provide further evidence for the dinoflagellate mitochondrial location of the *cox1 *and *cob *genes identified here, so all *D. baltica *gene fragments were aligned to their respective transcripts and conserved editing sites examined. In total, 786 bp of *cox1 *were comparable, and 352 bp of *cob *were comparable, from which 11 and 7 edited sites were identified, respectively. The nature of these edits was similar to that of other dinoflagellates (Table 1) and, significantly, all but three of the editing sites were conserved in other dinoflagellate mitochondria [22,35,40]. Overall, the characteristics of this editing are consistent with these genes and cDNAs being located in the dinoflagellate host mitochondria.

**Table 1:** Editing sites in the host mitochondrial mRNA of *cox1 *and *cob *in *Durinskia baltica*

Site Relative to *Cc *or *Pp*	DNA	RNA	Codon Position	Change aa	Conserved in
*cox1 *330	A	G	1st	I – V	*Pp *and *Cc*
*cox1 *351	T	C	1st	F – L	*Pp *and *Cc*
*cox1 *469	C	U	2nd	T – I	Unique^1^
*cox1 *481	C	U	2nd	S – F	*Pp *and *Cc*
*cox1 *495	T	C	1st	F – L	*Pp *and *Cc*
*cox1 *621	A	G	1st	I – V	*Pp *and *Cc*
*cox1 *691	A	G	2nd	Y – C	*Pp*
*cox1 *924	A	G	1st	I – V	*Pp *and *Cc*
*cox1 *952	T	C	2nd	L – S	*Pp *and *Cc*
*cox1 *1174	A	G	2nd	K – R	*Pp *and *Cc*
*cox1 *1180	A	G	2nd	N – S	*Pp *and *Cc*
*cob *782	T	C	2nd	V – A	*Pp*, *Cc*, *Pmic*, *Km*, *Pmin*, *Ps*, *At*
*cob *788	G	C	2nd	G – A	*Pp*, *Pmic*, *Km*, *Pmin*, *Ps*, *At*
*cob *861	C	U	3rd	Silent	*Pmic*, *Pmin*, *Km*
*cob *883	A	G	1st	I – V	*Pmic*, *Pmin*, *K.m.*
*cob *904	A	G	1st	T – A	*Pmic *and *Pmin*
*cob *1064	G	C	2nd	G – A	Unique^2^
*cob *1081	C	U	1st	H – V	Unique^2^

Column 1 is the site numbered according to genes from *Crypthecodinium cohnii *(*cox1*) or *Pfiesteria piscicida *(*cob*). Column 2 and 3 are pre-edited and post-edited states in *D. baltica*. Column 4 is the position within the codon. Column 5 is the amino acid change. Column 6 lists other dinoflagellates where the same editing is found. Abbreviations: *Pp, Pfiesteria piscicida*; *Cc*, *Crypthecodinium cohnii; Pmic*, *Prorocentrum micans*; *Prorocentrum minimum*; *Km*, *Karlodinium micrum*; *Ps*, *Pfiesteria shumwyae*; *At*, *Alexandrium tamarense*.

1 This change is absent in *Pp *and *Cc*.

2 These changes are absent in *Pp *and not sampled from *Cc*.

### Reduction and functional redundancy of mitochondria

Electron microscopy has shown that mitocondria exist in both the host and endosymbiont cytosolic compartments of *D. baltica *and *K. foliaceum *[11,12,16]. More recently diatom-derived genes for *cox1*, *cox3*, and *cob *have been shown to be expressed in *K. foliaceum *[9]. However, without comparable data from the host mitochondria it is impossible to determine whether the two organelles are functionally redundant, or have distributed functions between them. Here, we have shown that both organelles contain at least two genes with central functions in electron transport, *cox1 *and *cob*. Accordingly, these two species are unique among eukaryotes in having retained active, functional mitochondrial genomes from two distantly related eukaryotic lineages, the dinoflagellate host and the pennate diatom endosymbiont. Moreover, these organelles now appear to be at least partially functionally redundant, since both express genes for proteins in the electron transport chain.

Indeed, no characteristic of either host or endosymbiont mitochondrial genes or genomes has so far been shown to be significantly different from those of their dinoflagellate or diatom relatives, which points to the conclusion that neither organelle has been much affected by the presence of the other. It seems unlikely that the two mitochondria are retained due to functional differentiation, but their genetic redundancy may be related to spatial differentiation. If the membrane separating the host and the endosymbiont, which is thought to be derived from the diatom plasma membrane [20], were deficient in transporters to efficiently shuttle either substrates or products of mitochondrial reactions between the two compartments, then neither compartment could eliminate those functions without consequences. In other endosymbiotic events such difficulties would have been overcome or made irrelevant by the continued reduction of the endosymbiont and integration with the host. Whether the mitochondrion of *D. baltica *is a snapshot in the progression of events that will ultimately lead to its loss, or whether the process has been 'stuck' in some way is unknown. Similarly, although it is generally assumed that the endosymbiont will be reduced and the corresponding host feature retained, this does not need to be the case. With the already highly reduced and unusual nature of dinoflagellate mitochondrial genome [21,23,39], it is not unreasonable to hypothesize that the host organelle may be lost as easily as the relatively normal mitochondrion of the endosymbiont.

## Conclusion

We have shown that two related dinoflagellates, *D. baltica *and *K. foliaceum*, retain redundant genes in their host-derived and endosymbiont-derived mitochondrial genomes, including several genes related to electron transport. Host-derived genes are edited at the RNA level and subject to extensive recombination, as is expected for dinoflagellate mitochondria. All genes characterized have been shown to be expressed at the mRNA level, suggesting the two organelles overlap in function, making these unique among eukaryotes in retaining two partially redundant mitochondria with different evolutionary origins.

## Methods

### Culture conditions and nucleic acid extraction

Cultures of *Durinskia baltica *(*Peridinium balticum*) CS-38 were obtained from CSIRO Microalgae Supply Service (CSIRO Marine and Atmospheric Research Laboratories, Tasmania, Australia) and maintained in GSe medium at 22°C (12 : 12 light : dark cycle). Cultures of *Kryptoperidinium foliaceum *CCMP 1326 were obtained from the Provasoli-Guillard National Center for Culture of Marine Phytoplankton (West Boothbay Harbor, ME, USA) and maintained in F/2-Si medium under the above-mentioned conditions. Cultures were grown both with and without antibiotics to reduce the number of bacteria: 500 μg/ml penicillin G, 200 μg/ml ampicillin, 50 μg/ml streptomycin sulphate, and 50 μg/ml neomycin, modified from [19]. Cultures used in some molecular experiments did not contain antibiotics, while others did. Exponentially growing cells were harvested by centrifugation at 3,220 *g *for 5 min at 8°C, and the pellet was frozen and ground under liquid nitrogen. The total genomic DNA was extracted from about 100 mg of the ground cells using DNeasy Plant DNA isolation kit (Qiagen, Mississauga, ON). Total RNA was isolated using TRIzol Reagent (Invitrogen, Burlington, ON) from the pelleted cells following manufacturer's instructions, and it was treated with Deoxyribonuclease I (Invitrogen). PCR was carried out using PuReTaq (Amersham Biosciences, Baie d'Urfé, QC) and long range PCR using Elongase Enzyme Mix (Invitrogen). RT-PCR was carried out using SuperScript III One-Step System with Platinum Taq DNA Polymerase (Invitrogen).

### Amplification and sequencing of mitochondrial genes and cDNAs

From genomic DNA of *D. baltica*, for amplification of the endosymbiont genes we used the following primers: for *LSUrRNA *gene, 5'-TTCTGCGAAATCTATTKAAGTAGAGCG-3' and 5'-CYGGCGTACCTTTTATCCRTTGMGC-3'; for *cob *gene, 5'-CCCTTACAGCAATTCCATTCGGAGGTCAAA-3' and 5'-TTCGCCCTTCTGGAATACAATTATCAGGAT-3'; for *cox3 *gene, 5'-TTACAGGTGGTGTTCTTTATATGCACAAAA-3' and 5'-AGCCGAAGTGGTGGGGTATTTGTTGAGTGGT-3'; for *cox2 *gene, first we used the following two degenerate primers, 5'-ATCGGGCATCAGTGGTAYTGGWSNTAYGA-3' and 5'-GTTTATCCCGCAGATYTCNSWRCAYTGNCC-3' and later the following specific primers, 5'-GTATTGGAGGTACGAGATTTCGGACTTTGA-3' and 5'-CGGAGCACTGACCAAAGAACATACCCACA-3'; for *cox1 *gene, 5'-GTTGTTACCCACCTTCTCTTTTACTACTGAT-3' and 5'-GCAACAACGTAATAAGTATCGTGAGGAGCA-3'. For amplification of the host genes in *D. baltica *from genomic DNA, and all the *cox1 *products containing an insert, we used the following primers: for *cox1*, first we used the following two primers previously described [35], 5'-AAAAATTGTAATCATAAACGCTTAGG-3' and 5'-TGTTGAGCCACCTATAGTAAACATTA-3', and later the following two specific primers, 5'-GCACTTCTTTCATGAGTTTATCACCTTCAAG-3' and 5'-TTCTGAGCTGTAACAATGGCGGATTCCCA-3'; for *cob*, initially the following two primers were used, 5'-GGGGTGCTACGGTTATTACGAACCTACTA-3' and 5'-TGCCTAACAAAAATGCAGGATTCATAGTCT-3', and later the following primer was used to amplify the 3' end of the gene in 3' RACE using RLM-RACE kit (Ambion, Austin, TX, USA) following the manufacturer's instructions, 5'-GCATTAGAAGCTTGTGCATTACTTACTCCT-3'. From genomic DNA of *K. foliaceum*, for amplification of the endosymbiont genes we used the following primers: for *LSUrRNA *gene, 5'-AACAGACAGTCCATGAGTGCTAAGATTCAT-3', and 5'-CACACAGAATTACCGGATCACTATAACCGA-3'; for *cox2 *gene, we first used the following two degenerate primers, 5'-GGGCATCAGTGGTATTGGWSNTAYGARWW-3' and 5'-GTTTATCCCGCAGATYTCNSWRCAYTGNCC-3', and later the following two specific primers, 5'-GGGCATCAGTGGTATTGGTGGTACGAAAT-3' and 5'-GTTTATCCCGCAGATTTCGCTGCACTGGCC-3'. For amplification of the host *cox1 *in *K. foliaceum *from total RNA, we used the two previously described primers [35], 5'-AAAAATTGTAATCATAAACGCTTAGG-3' and 5'-TGTTGAGCCACCTATAGTAAACATTA-3', using RT-PCR. Transcripts of all the genes were characterized by RT-PCR using the same primers, and all these amplifications were carried out with controls lacking RT enzyme, from which no products were acquired.

We also used three pairs of dinoflagellate-specific primers to search for the host *cob *in *K. foliaceum*, which were based on the most conserved regions of this gene found in dinoflagellate mitochondria. Two pairs of these primers were tested successfully to amplify this gene from *D. baltica *(data not shown). However, no product was obtained with any of these primers from the total DNA or RNA extracted from *K. foliaceum *used in PCR and RT-PCR respectively.

All PCR and RT-PCR products were gel purified and cloned using pCR 2.1 TOPO Cloning kit (Invitrogen). In each case, several clones were sequenced on both strands using BigDye terminator chemistry. New sequences have been deposited into GenBank as accessions EF434607–EF434629.

### Phylogenetic analyses

The conceptual translations of new *cox1*, *cox2*, *cox3*, and *cob*, and DNA sequences for *LSUrRNA *from *D. baltica *and *K. foliaceum *were aligned with homologues from public database using ClustalX 1.83.1 [41] under the default gap opening and gap extension penalties and the alignments edited manually. *Phaeodactylum tricornutum *homologues were kindly provided by Marie-Pierre Oudot-Le Secq from the *P. tricornutum *genome sequencing project [42]. Phylogenetic analyses were carried out including a diversity of eukaryotes to determine the overall position of new sequences, and subsequently restricted to homologues from chromalveolate taxa (dinoflagellates, apicomplexans, ciliates, heterokonts, haptophytes, and cryptomonads), since both the host and endosymbiont are thought to be members of this supergroup [43]. No *LSUrRNA *sequences for dinoflagellates or apicomplexans were included since these genes are fragmented, only partially described to date, and highly divergent, so ciliates alone represent alveolates. These alignments consisted of 52, 20, 20, and 26 amino acid sequences, and 17 DNA sequences with 378, 109, 251, 372, and 1304 unambiguously aligned sites for *cox1*, *cox2*, *cox3*, *cob*, and *LSUrRNA*, respectively. For *cox1 *and *cob *sequences, several alignment alternatives were attempted, which were independently followed by phylogenetic analyses. These alternative alignments differed only in the inclusion or exclusion of missing sites. In order to make use of all the recovered sequence data, the alignments of *cox1 *and *cob *that were analysed included some missing data and either the 5' or 3' ends of certain *D. baltica *or *K. foliaceum *genes. Phylogenies of *cox1 *and *cob *were also performed using excluding either host or endosymbiont genes, resulting in no significant differences (not shown). All alignments are available upon request.

Phylogenetic trees were inferred using maximum likelihood and distance methods. The proportion of invariable sites (i) and shape parameter alpha (α) with 8 variable rate categories were estimated from the data with PhyML 2.4.4 [44] under the Whelan and Goldman (WAG) model of substitution for *cox1*, *cox2*, *cox3*, and *cob *phylogenies, and under General Time-Reversible (GTR) model of substitution for *LSUrRNA *phylogeny with the frequency of amino acid or nucleotide usage calculated from the data. The i and α parameters estimated from the data were 0.000, 0.057, 0.004, 0.010, and 0.113, and 1.002, 1.297, 1.186, 1.402 and 1.002 for *cox1*, *cox2*, *cox3*, *cob*, and *LSUrRNA*, respectively. For all five data sets 1,000 bootstrap replicates were analyzed using PhyML. For distance trees, distances were calculated using TREE-PUZZLE 5.2 [45] with 8 variable rate categories and invariable sites. The i and α parameters were estimated by TREE-PUZZLE to be 0.00, 0.05, 0.00, 0.00, and 0.10, and 0.94, 1.18, 1.03, 1.12, and 0.91 for *cox1*, *cox2*, *cox3*, *cob*, and *LSUrRNA*, respectively. Trees were constructed by weighted neighbor-joining using WEIGHBOR 1.0.1a [46]. Distance bootstrapping of 1,000 replicates was carried out using PUZZLEBOOT (shell script by A. Roger and M. Holder).

## Competing interests

The author(s) declares that there are no competing interests.

## Authors' contributions

BI characterized new molecular sequences, carried out phylogenetic analysis and drafted the manuscript. PJK conceived of the study, participated in its design, participated in sequence analysis, and helped draft the manuscript. Both BI and PJK read and approved the final manuscript.
